# Clinical and microbiological characteristics and follow-up of invasive *Listeria monocytogenes* infection among hospitalized patients: real-world experience of 16 years from Hungary

**DOI:** 10.1186/s12866-024-03478-z

**Published:** 2024-09-06

**Authors:** Rebeka Kiss, Bence Marosi, Dorina Korózs, Borisz Petrik, Botond Lakatos, Bálint Gergely Szabó

**Affiliations:** 1 Faculty of Medicine, Semmelweis University, H-1085 Ulloi ut 26, Budapest, Hungary; 2 South Pest Central Hospital, National Institute of Hematology and Infectious Diseases, H-1097 Albert Florian ut 5-7, Budapest, Hungary; 3 Doctoral School of Clinical Medicine, Semmelweis University, H-1085 Ulloi ut 26, Budapest, Hungary; 4 Department of Haematology and Internal Medicine, Division of Infectology, Semmelweis University, Albert Florian ut 5‑7, Budapest, H-1097 Hungary

**Keywords:** *Listeria monocytogenes*, Listeriosis, Neurolisteriosis, Bloodstream-infection, Sepsis

## Abstract

**Purpose:**

Invasive *Listeria monocytogenes* infection is rare, but can lead to life-threatening complications among high-risk patients. Our aim was to assess characteristics and follow-up of adults hospitalized with invasive *L. monocytogenes* infection.

**Methods:**

A retrospective observational cohort study was conducted at a national referral center between 2004 and 2019. Patients with proven invasive listeriosis, defined by the European Centre for Disease Prevention and Control criteria, were included. Data collection and follow-up were performed using the hospital electronic system, up until the last documented visit. The primary outcome was in-hospital all-cause mortality, secondary outcomes included residual neurological symptoms, brain abscess occurrence, and requirement for intensive care unit (ICU) admission.

**Results:**

Altogether, 63 cases were identified (57.1% male, median age 58.8 ± 21.7 years), and 28/63 developed a complicated disease course (44.4%). At diagnosis, 38/63 (60.3%) presented with sepsis, 54/63 (85.7%) had central nervous system involvement, while 9/63 (14.3%) presented with isolated bacteremia. Frequent clinical symptoms included fever (53/63, 84.1%), altered mental state (49/63, 77.8%), with immunocompromised conditions apparent in 56/63 (88.9%). *L. monocytogenes* was isolated from blood (37/54, 68.5%) and cerebrospinal fluid (48/55, 87.3%), showing in vitro full susceptibility to ampicillin and meropenem (100% each), gentamicin (86.0%) and trimethoprim/sulfamethoxazole (97.7%). In-hospital all-cause mortality was 17/63 (27.0%), and ICU admission was required in 28/63 (44.4%). At discharge, residual neurological deficits (11/46, 23.9%) and brain abscess formation (6/46, 13.0%) were common.

**Conclusion:**

Among hospitalized adult patients with comorbidities, invasive *L. monocytogenes* infections are associated with high mortality and neurological complications during follow-up.

**Supplementary Information:**

The online version contains supplementary material available at 10.1186/s12866-024-03478-z.

## Introduction

Human listeriosis is a zoonotic infection mainly caused by *Listeria monocytogenes* (abbreviated as *L. monocytogenes*), a facultative intracellular Gram positive rod widely spread in the environment due to its high resistance to external exposures, such as low temperature [1]. As a result, *L. monocytogenes* poses a significant hazard in the food industry as human infection is primarily caused by ingesting soil-contaminated vegetables, fruits, water, dairy products, or animal meat [2–6]. Asymptomatic carriage of *L. monocytogenes* can occur in 0.8–3.4%,which may also contribute to disease transmission [7]. In healthy adults, ingestion of 1.0 × 10^9^ bacteria could lead to moderate to severe acute gastroenteritis, while in individuals with predisposing factors, even a lower bacterial concentration (1.0 × 10^2^–10^4^) could result in severe and invasive infections such as sepsis, meningitis, miscarriage or perinatal infections [1, 8–10]. The diagnosis of invasive listeriosis is established through microbiological examination by culturing or nucleic acid amplification of relevant clinical samples (e.g. blood, cerebrospinal fluid), and the first-line treatment typically includes ampicillin or penicillin-G, with or without gentamicin [11–14].

In Hungary, human listeriosis was first identified in 1965, and over the next 25 years, almost half of the 90 documented cases occurred in pregnant or newborn patients, with a mortality rate as high as 45% [15]. In the past decade, the European Centre for Disease Prevention and Control has reported 24 to 39 new cases of human listeriosis annually, but no clinical study about invasive listeriosis has been conducted in Hungary so far [8]. Therefore, our aim was to assess the clinical and microbiological characteristics of invasive *Listeria monocytogenes* infections among hospitalized patients during a 16-year period, with a particular emphasis on adverse clinical outcomes and in vitro resistance patterns of isolates.

## Methods

### Study design

We conducted a retrospective observational cohort study of patients hospitalized for proven invasive listeriosis during a 16-year period from January, 2004 to December, 2019 at South Pest Central Hospital, National Institute of Hematology and Infectious Diseases in Budapest, Hungary (SPCH–NIHI). This tertiary referral center has ≥ 100 dedicated beds for infectious diseases. The study protocol adhered to the recommendations of the Helsinki Declaration and national ethical standards, and was approved by the Institutional Review Board of South Pest Central Hospital, National Institute of Hematology and Infectious Diseases in Budapest, Hungary (IKEB/37/2016). This type of study does not require written informed consent from patients according to national regulations.

### Patient identification and selection

Patients hospitalized with a probable or proven clinical case during the study period were eligible for inclusion. Patients were identified using the prospective data-archiving electronic system of the hospital, utilizing 10th International Classification of Diseases codes consistent with admission and discharge diagnoses of listeriosis (A32, A41, P37). To overcome selection bias, a secondary query was performed from the archiving system of the Central Microbiology Laboratory of our center for all *Listeria* spp. isolated from clinical samples during the study period. All eligible cases were retrospectively evaluated. Included patients were subgrouped based on the development of a complicated disease course, into two subgroups: (I) Uncomplicated disease course and (II) Complicated disease course (see Tables 1, 2 and 3). Complicated disease was defined by the next: (1) in-hospital death or (2) occurrence of L. monocytogenes brain abscess or (3) presence of residual neurological symptoms at discharge. Patients were included if invasive listeriosis was proven microbiologically (see definitions later). Exclusion criteria were as follows: (1) patient data were unavailable in the electronic system, (2) the final diagnosis did not correspond to invasive listeriosis, (3) patient death or transfer to another hospital within ≤ 72 h of admission. Using the database, follow-up was conducted for all included patients until hospital discharge or the last available outpatient visit (follow-up was completed on June, 2023).

**Table 1:** Demographic and baseline characteristics of patients with invasive *Listeria monocytogenes* infection in the cohort, grouped by disease course

Parameter	Full cohort (*n* = 63)	Uncomplicated disease course (*n* = 35)	Complicated disease course (*n* = 28)	*p* value
Age (years, median ± IQR, min–max)	58.8 ± 21.7 (0.8–86.4)	55.0 ± 31.3 (1.6–81.7)	63.5 ± 17.0 (0.8–86.4)	0.09
Male sex (n, %)	36 (57.1)	19 (54.3)	17 (60.7)	0.61
Admission from another hospital (n, %)	31 (49.2)	14 (40.0)	17 (60.7)	0.10
Calendar number of admission month (median ± IQR, min–max)	7 ± 6 (1–12)	8 ± 4 (1–12)	6 ± 9 (1–12)	0.42
Potential source of transmission identified (n, %)	3 (4.8)	3 (8.6)	0 (0.0)	0.11
Comorbidities (n, %):				
- Essential hypertension - Chronic heart disease - Chronic peripheral vascular disease - Chronic cerebrovascular disease - Chronic lung disease - Chronic kidney disease - Chronic liver disease - Diabetes mellitus - Active onco-hematological malignancy - Systemic autoimmune disease - HIV infection - Immunocompromising conditions - Chronic alcohol consumption	37 (58.7) 27 (42.9) 26 (41.3) 27 (42.9) 15 (23.8) 8 (12.7) 22 (34.9) 19 (30.2) 11 (17.5) 19 (30.2) 2 (3.2) 56 (88.9) 17 (27.0)	17 (48.6) 10 (28.6) 10 (28.6) 12 (34.3) 7 (20.0) 1 (2.9) 11 (31.4) 8 (22.9) 4 (11.4) 12 (34.3) 1 (2.9) 28 (80.0) 7 (20.0)	20 (71.4) 17 (60.7) 16 (57.1) 15 (53.6) 8 (28.6) 7 (25.0) 11 (39.3) 11 (39.3) 7 (25.0) 7 (25.0) 1 (3.6) 28 (100.0) 10 (35.7)	0.07 **0.01** **0.02** 0.12 0.43 **0.01** 0.52 0.16 0.16 0.43 0.87 **0.01** 0.16
Predisposing factors for invasive listeriosis (n, %):				
- Age ≤ 1 year - ≥64 years of age - Long-term proton pump inhibitor therapy - Long-term systemic corticosteroid treatment - Other immunomodulatory therapies - Antibiotic use in the last 3 months - Immunotherapy/chemotherapy in the last 6 months - Conditions with iron overload - Haematopoietic stem cell transplantation - Colonoscopy in the last 3 months	1 (1.6) 27 (42.9) 25 (39.7) 19 (30.2) 13 (20.6) 10 (15.9) 4 (6.3) 1 (1.6) 1 (1.6) 4 (6.3)	0 (0.0) 13 (37.1) 11 (31.4) 11 (31.4) 8 (22.9) 8 (22.9) 2 (5.7) 0 (0.0) 0 (0.0) 3 (8.6)	1 (3.6) 14 (50.0) 14 (50.0) 8 (28.6) 5 (17.9) 2 (7.1) 2 (7.1) 1 (3.6) 1 (3.6) 1 (3.6)	n.a. 0.31 0.13 0.81 0.63 0.09 0.82 0.26 0.26 0.42
Number of comorbidities (per patient, median ± IQR, min–max)	4.0 ± 3.0 (0.0–9.0)	3.0 ± 3.0 (0.0–9.0)	5.0 ± 3.0 (1.0–9.0)	**< 0.01**
Number of predisposing factors (per patient, median ± IQR, min–max)	4.0 ± 3.0 (0.0–10.0)	4.0 ± 3.5 (0.0–9.0)	4.5 ± 2.0 (3.0–10.0)	0.23
Charlson score (median ± IQR, min–max)	5.0 ± 4.0 (0.0–12.0)	4.0 ± 4.0 (0.0–11.0)	6.0 ± 3.3 (1.0–12.0)	**< 0.01**

HIV: human immunodeficiency virus, IQR: interquartile range, n.a.: not applicable

**Table 2:** Clinical characteristics of patients with invasive *Listeria monocytogenes* infection in the cohort at diagnosis, grouped by disease course

Parameter	Full cohort (*n* = 63)	Uncomplicated disease course (*n* = 35)	Complicated disease course (*n* = 28)	*p* value
Changes in core body temperature (n, %)				
- Fever (≥ 38.0 °C) - Hypothermia (< 36.0 °C) - Euthermia	53 (84.1) 1 (1.6) 9 (14.3)	33 (94.3) 1 (2.9) 1 (2.9)	20 (71.4) 0 (0.0) 8 (28.6)	**0.01** n.a. **< 0.01**
Musculoskeletal symptoms (n, %)				
- Joint and muscle pain - Back pain	4 (6.3) 6 (9.5)	2 (5.7) 6 (17.1)	2 (7.1) 0 (0)	0.82 **0.02**
Neurological symptoms (n, %)				
- Altered mental state - Signs of meningeal irritation - Focal neurological symptoms - Headache	49 (77.8) 38 (60.3) 35 (55.6) 30 (47.6)	23 (65.7) 20 (57.1) 13 (37.1) 23 (65.7)	26 (92.9) 18 (64.3) 22 (78.6) 7 (25.0)	0.53 0.57 **< 0.01** **< 0.01**
Gastrointestinal symptoms (n, %)				
- Vomiting - Diarrhoea - Singultus	22 (34.9) 18 (28.6) 6 (9.5)	15 (42.9) 11 (31.4) 2 (5.7)	7 (25.0) 7 (25.0) 4 (14.3)	0.14 0.57 0.26
Other symptoms (n, %)				
- Skin lesion - Hepatomegaly - Splenomegaly - Conjunctival injection - Acute bleeding - Acute respiratory failure - Acute renal failure	13 (20.6) 29 (46.0) 12 (19.0) 13 (20.6) 17 (27.0) 32 (50.8) 8 (12.7)	5 (14.3) 14 (40.0) 9 (25.7) 4 (11.4) 6 (17.1) 9 (25.7) 0	8 (28.6) 15 (53.6) 3 (10.7) 9 (32.1) 11 (39.3) 23 (82.1) 8 (28.6)	0.16 0.28 0.13 **0.04** **0.04** **< 0.01** **< 0.01**
Clinical manifestations of infection (n, %)				
- Meningitis / meningoencephalitis - Isolated bacteraemia - Sepsis	54 (85.7) 9 (14.3) 38 (60.3)	29 (82.9) 6 (17.1) 15 (49.2)	25 (89.3) 3 (10.7) 23 (82.1)	0.47 0.24 **< 0.01**
Time from symptom onset to hospital admission (days, median ± IQR, min-max)	2.0 ± 3.0 (0.0–15.0)	2.0 ± 2.0 (0.0–15.0)	3.0 ± 3.0 (0.0–11.0)	0.74

IQR: interquartile range, n.a.: not applicable

**Table 3:** Laboratory, microbiological and imaging characteristics of patients with invasive *Listeria monocytogenes* infection in the cohort at diagnosis, grouped by disease course

Parameter	Full cohort (*n* = 63)	Uncomplicated disease course (*n* = 35)	Complicated disease course (*n* = 28)	*p* value
Blood laboratory parameters at diagnosis (median ± IQR, min-max)				
- White blood cell count (x10^9^/l) - Absolute neutrophil granulocyte count (x10^9^ l) - Absolute lymphocyte count (x10^9^/l) - Absolute monocyte count (x10^9^/l) - Haemoglobin (g/l) - Platelet count (x10^9^/l) - Serum procalcitonin (ng/ml) - Serum C-reactive protein (mg/L) - Serum lactate dehydrogenase (IU/l) - Serum glutamate oxaloacetate transaminase (IU/l) - Serum glutamate pyruvate transaminase (IU/l) - Serum gamma-glutamyl transferase (IU /l) - Serum alkaline phosphatase (IU/l) - Serum urea (mmol/l) - Serum creatinine (µmol/l) - Serum glucose (mmol/l) - Serum albumin (g/l) - Serum total protein (g/l) - Serum total bilirubin (µmol/l)	11.8 ± 6.8 (2–38) 9.9 ± 6.6 (1.8–20.9) 0.7 ± 0.6 (0.2–14.8) 0.6 ± 0.6 (0.1–2.8) 118.0 ± 36.0 (63.0–163.0) 167.5 ± 108.5 (44.0–499.0) 1.4 ± 2.5 (0–164.7) 137.0 ± 221.5 (5.0–482.0) 505.0 ± 260.0 (143.0–1267.0) 33.5 ± 34.5 (3.0–702.0) 26.0 ± 35.8 (4.0–487.0) 82.0 ± 106.5 (11.0–734.0) 210.0 ± 161.0 (92.0–617.0) 5.3 ± 4.2 (1.2–33.5) 76.5 ± 45.5 (7.8–237.0) 7.5 ± 3.1 (2.5–29.4) 33.5 ± 8.5 (20.0–45.0) 60.0 ± 9.8 (40.0–80.0) 14.6 ± 13.7 (3.8–162.7)	12.5 ± 5.8 (3.1–38.5) 9.3 ± 5.9 (1.8–20.9) 0.7 ± 0.7 (0.3–14.8) 0.7 ± 0.6 (0.1–2.8) 121.0 ± 31.0 (85.0–163.0) 198.5 ± 93.3 (54.0–499.0) 1.4 ± 1.8 (0.0–164.7) 107.0 ± 205.0 (5.0–482.0) 454.5 ± 198.8 (143.0–1263.0) 23.5 ± 27.3 (3.0–702.0) 22.5 ± 29.8 (4.0–487.0) 60.0 ± 90.0 (11.0–194.0) 193.0 ± 173.0 (103.0–501.0) 5.0 ± 3.9 (1.2–14.3) 71.0 ± 49.8 (7.8–153.0) 7.3 ± 2.5 (3.3–29.4) 35.0 ± 13.5 (23.0–45.0) 60.5 ± 9.0 (45.0–80.0) 12.1 ± 14.1 (4.2–162.7)	11.4 ± 6.3 (2.4–23.3) 9.9 ± 8.2 (2.0–18.9) 0.7 ± 0.7 (0.2–4.2) 0.6 ± 0.8 (0.1–2.3) 114.0 ± 38.3 (63.0–156.0) 131.5 ± 113.5 (44.0–311.0) 1.9 ± 4.1 (0.2–26.7) 139.5 ± 187.0 (22.0–366.0) 595.0 ± 383.0 (290.0–1267.0) 44.5 ± 48.8 (14.0–166.0) 30.5 ± 30.8 (8.0–151.0) 98.0 ± 235.0 (15.0–734) 226.5 ± 196.0 (92.0–617.0) 5.5 ± 4.2 (1.2–33.5) 86.0 ± 49.0 (12–237) 7.8 ± 5.0 (2.5–18.1) 32.0 ± 6.3 (20.0–44.0) 59.5 ± 13.3 (40.0–74.0) 15.3 ± 14.4 (3.8–140.3)	0.21 0.41 0.32 0.84 0.32 **0.05** 0.56 0.74 **0.01** 0.6 0.7 **0.02** 0.26 0.07 0.06 0.95 0.17 0.51 0.73
CSF laboratory parameters at diagnosis (median ± IQR, min-max)				
- CSF white blood cell count (/mm^3^) - CSF neutrophil granulocytes (%) - CSF lymphocytes (%) - CSF monocytes (%) - CSF lymphocyte/neutrophil granulocyte ratio - CSF glucose (mmol/l) - CSF protein (g/l)	410.0 ± 783.0 (0.0–5000.0) 54.0 ± 49.0 (0.0–89.0) 46.0 ± 52.5 (10.0–100) 0.0 ± 0.0 (0.0–10.0) 0.9 ± 1.8 (0.1–49.0) 2.2 ± 2.1 (0.2–9.1) 2.3 ± 1.8 (0.3–15.0)	465.0 ± 937.5 (0.0–5000.0) 43.0 ± 60.5 (2.0–86.0) 55.0 ± 63.0 (10.0–98.0) 0.0 ± 1.5 (0.0–10.0) 1.4 ± 8.0 (0.1–49.0) 2.2 ± 2.0 (0.2–5.6) 2.2 ± 1.6 (0.3–6.0)	400.0 ± 520.0 (11.0–2400.0) 65.0 ± 41.3 (0.0–89.0) 32.5 ± 45.0 (11.0–100.0) 0.0 ± 0.0 (0.0–10.0) 0.3 ± 1.0 (0.1–24.0) 1.8 ± 2.5 (0.3–9.1) 2.4 ± 4.3 (0.4–15.0)	0.18 0.46 0.55 0.89 0.70 0.47 0.09
Blood microbiological parameters at diagnosis (n, %)				
- Blood cultures acquired - True bacteremia *	54 (85.7) 37 (68.5)	28 (80.0) 18 (64.3)	26 (92.9) 19 (73.1)	0.15 0.19
CSF microbiological parameters at diagnosis (n, %)				
- CSF sample acquired - Positive CSF **	55 (87.3) 48 (87.3)	30 (85.7) 27 (90.0)	25 (89.3) 21 (84.0)	0.67 0.84
Cranial CT scan at diagnosis (n, %)				
- CT scan performed - Abnormal CT scan	41 (65.1) 31 (75.6)	18 (51.4) 14 (77.8)	23 (82.1) 17 (73.9)	**0.01** 0.10
Cranial MRI scan at diagnosis (n, %)				
- MRI scan performed - Abnormal MR scan	21 (33.3) 14 (66.7)	10 (28.6) 6 (60.0)	11 (39.3) 8 (72.7)	0.37 0.28
EEG at diagnosis (n, %)				
- EEG performed - Abnormal EEG	28 (44.4) 28 (100.0)	10 (28.6) 10 (100.0)	18 (64.3) 18 (100.0)	**< 0.01** 1.0

CSF: cerebrospinal fluid, CT: computed tomography, EEG: electroencephalography, MRI: magnetic resonance imaging, IQR: interquartile range

* *L. monocytogenes alone* was isolated from blood cultures in all cases without any other pathogen

** Presence of *L. monocytogenes* was confirmed from CSF in all cases without any other pathogen

### Data collection and definitions

Anonymized data were manually entered into an electronic data collection spreadsheet, standardized by the lead investigator. Collected data included: (1) age and sex, (2) comorbidities and predisposing factors for invasive listeriosis, (3) clinical presentation, (4) microbiological and laboratory examinations, (5) types of empirical and targeted antibiotics and supportive therapy, (6) outcomes. Baseline data were recorded on the day of diagnosis.

Invasive listeriosis was defined according to the case definition of the European Centre for Disease Prevention and Control [16]. Microbiological criteria were as follows: 1) presence of *Listeria* spp. detected through culturing methods or polymerase chain reaction (PCR) in a sample obtained from a physiologically sterile site (blood, cerebrospinal fluid, synovium, pericardial, pleural, or peritoneal fluid, etc.) or non-sterile site (placenta, amniotic fluid, meconium, etc.). Clinical criteria included: (1) meningitis, encephalitis, (2) sepsis and febrile influenza-like syndrome, (3) certain local infections (arthritis, endocarditis, endophthalmitis, abscess of unknown etiology), (4) pregnancy (miscarriage, stillbirth, preterm birth) and neonatal (granulomatosis infantiseptica, skin and mucocutaneous lesions of unknown origin) complications. Isolation and identification of *L. monocytogenes* were performed using classical culturing and Gram staining, matrix-assisted laser desorption/ionization time-of-flight mass spectrometry (MALDI Biotyper, Bruker, USA), cerebrospinal fluid (CSF) PCR (BioFire FilmArray Meningitis/Encephalitis Panel, bioMérieux, France), and latex agglutination (Listeria Latex Test, Microgen Bioproducts, UK). In vitro antibiotic susceptibility testing followed the recommendations of the European Committee on Antimicrobial Susceptibility Testing (EUCAST) current at the time of diagnosis [17]. Microbiological diagnostics were conducted at the Central Microbiology Laboratory of our hospital.

We defined adequate empirical antibiotic therapy against *L. monocytogenes* as: (1) a patient with suspected invasive listeriosis receiving ≥ 1 empirically selectable antibiotic within 24 h of diagnosis, in the appropriate dose and formulation, according to international recommendations, (2) no need for antimicrobial escalation before the final microbiological result was known, or (3) the isolate subsequently detected by culture showed in vitro antibiotic susceptibility to the chosen agent(s), or if presence of the bacterium was not confirmed by culture, intrinsic antibiotic resistance was not suspected [18]. Inadequate empirical antibiotic therapy was considered if ≥ 1 of these criteria were not met. Potential choices for adequate antibiotics against *Listeria* spp. included amoxicillin, ampicillin, meropenem, linezolid, trimethoprim/sulfamethoxazole, and vancomycin. Escalation of antibiotic therapy was defined as a change in empirical choice to broaden the spectrum of activity before microbiological results. Targeted antibiotic therapy was defined as continuing or de-escalating empirical therapy based on microbiological results. The American College of Chest Physicians/Society of Critical Care Medicine pediatric and adult Systemic Inflammatory Response Syndrome (SIRS) criteria were used to define sepsis [19]. Charlson index was used to define comorbidity burden [20]. Long-term systemic corticosteroid therapy was defined as ≥ 3 months of ≥ 15 mg/day of prednisone-equivalent dose of systemic glucocorticoids. Immunocompromised conditions were defined as congenital immunodeficiencies, active oncohematological malignancy, solid organ or hematopoietic stem cell transplantation, ongoing immunotherapy/chemotherapy or immunomodulatory therapy, systemic autoimmune diseases, functional or anatomical asplenia, uncontrolled HIV infection, and end-stage liver cirrhosis.

### Clinical outcomes

The primary outcome was in-hospital all-cause mortality. Secondary outcomes were: (1) residual neurological symptoms, (2) occurrence of brain abscess, (3) need for intensive care unit (ICU) admission due to *L. monocytogenes* infection. All outcomes were assessed at hospital discharge or the last available outpatient visit.

### Statistical analysis

Continuous and categorical variables are presented as median ± interquartile range (IQR) with minimum–maximum values and absolute numbers of cases (n) and percentage relative frequency (%), respectively. Mann-Whitney U-test and Fisher’s exact test were used for statistical comparisons. Statistical significance was determined at a two-sided p-value < 0.05. Calculations were performed using IBM SPSS Statistics 23.

## Results

### Demographic and baseline characteristics

During the study period, 63 cases of invasive listeriosis were identified. The demographic and baseline patient characteristics are shown in Table 1. The median age in the cohort was 58.8 ± 21.7 years, and the male-to-female ratio was balanced between subgroups. A complicated disease course affected 44.4% (28/63) of patients. Most patients were admitted during the summer months, but no outbreaks were identified. All patients were admitted from the community, no nosocomial acquisitions were documented. Common comorbidities included immunocompromised conditions (56/63, 88.9%), essential hypertension (37/63, 58.7%), and chronic heart and cerebrovascular diseases (27/63, 42.9% each). Patients with subsequent complications had a significantly higher median Charlson score (6.0 ± 3.3 vs. 4.0 ± 4.0, *p* < 0.01). Predisposing factors for invasive listeriosis were identified in 59/63 (93.7%) cases, with most being ≥ 65 years of age (27/63, 42.9%), long-term proton pump inhibitor use (25/63, 39.7%), and systemic corticosteroid treatment (19/63, 30.2%).

### Clinical characteristics

Clinical characteristics are presented in Table 2. The median symptom onset before admission was 2.0 ± 3.0 days, with fever (53/63, 84.1%) and confusion (49/63, 77.8%) being the most frequent symptoms. Signs of meningeal irritation were observed in 60.3% (38/63) of cases. Euthermia (1/35, 2.9% vs. 8/28, 28.6%, *p* < 0.01), focal neurological signs (13/35, 37.1% vs. 22/28, 78.6%, *p* < 0.01), conjunctival injection (4/35, 11.4% vs. 9/28, 32.1%, *p* = 0.04), and the development of acute respiratory failure (9/35, 25.7% vs. 23/28, 82.1%, *p* < 0.01), shock (7/35, 20.0% vs. 16/28, 57.1%, *p* < 0.01), hemorrhage (6/35, 17.1% vs. 11/28, 39.3%, *p* = 0.04), or acute renal failure (0/35, 0.0% vs. 8/28, 28.6%, *p* < 0.01) were more prevalent in the complicated disease subcohort. Invasive listeriosis predominantly manifested as central nervous system infection (54/63, 85.7%), isolated bacteremia occurred in 9/63 (14.3%). Sepsis was documented in 60.3% of cases (38/63).

### Laboratory and imaging characteristics

Laboratory and imaging characteristics are provided in Table 3. A statistically significant difference was observed between the two subcohorts regarding initial platelet count (198.5 ± 93.3 × 10^9^/l vs. 131.5 ± 113.5 × 10^9^/l, *p* = 0.05), serum lactate dehydrogenase levels (454.5 ± 198.8 IU/l vs. 595.0 ± 383.0 IU/l, *p* = 0.01), and gamma-glutamyl transferase levels (60.0 ± 90.0 IU/l vs. 98.0 ± 235.0 IU/l, *p* = 0.02). During CSF analysis, hyperproteinorrachia (2.3 ± 1.8 g/l) with normoglycorrachia (2.2 ± 2.1 mmol/l) and pleiocytosis (410.0 ± 783.0 WBC/mm^3^) were the typical findings. Cranial CT and MRI scans were obtained in 65.1% (41/63) and 33.3% (21/63), revealing intracranial abnormalities in 75.6% (31/41) and 66.7% (14/21) of cases, respectively. The most common locations of abnormalities were diffuse subcortical (29/63, 46.0%), brainstem (5/63, 7.9%), frontal (4/63, 6.4%), parietal (4/63, 6.4%), temporal (1/63, 1.6%), and occipital (1/63, 1.6%). The rates of abnormalities detected with CT and MRI scans were statistically similar between the two subcohorts. Electroencephalography (EEG) was performed in 28/63 cases (44.4%), all of which yielded abnormal results. Fifteen patients (53.6%) exhibited diffuse cortical alterations, in 7/28 (25.0%) cases left hemisphere alterations, and in 3/28 (10.7%) cases right hemisphere alterations were detected.

### Microbiological characteristics

Microbiological characteristics are summarized in Table 3. Blood cultures were drawn in 54/63 cases (85.7%), with 68.5% (37/54) testing positive for *L. monocytogenes*. CSF samples were cultured in 55/63 cases (87.3%), yielding positive results in 48/55 (87.3%). CSF Gram staining findings were documented in 12/55 cases (21.8%), with 66.7% (8/12) reporting Gram positive rods, 25.0% (3/12) reporting Gram positive cocci, and 8.3% (1/12) reporting Gram negative rods. In 1/63 case (1.6%), *L. monocytogenes* was also cultured from tracheal secretion. Routine culture of *Listeria* spp. in feces was not conducted, but it was attempted once upon clinician request, yielding a negative result.

In vitro antibiotic susceptibility testing of *L. monocytogenes* isolates is shown in Table 4. Apart from penicillin (2/15, 13.3% moderately susceptible), isolates demonstrated full in vitro susceptibility to all tested beta-lactam antibiotics. Additionally, 100% in vitro susceptibility was observed for levofloxacin, erythromycin, tetracycline, rifampicin, chloramphenicol, and vancomycin. In vitro resistance to trimethoprim/sulfamethoxazole (1/44, 2.3%), ciprofloxacin (1/14, 7.1%), and clindamycin (1/3, 33.3%) was detected in 3 strains.

**Table 4:** Results of in vitro antibiotic susceptibility testing of isolates from clinical specimens of patients with invasive *Listeria monocytogenes* infection in the cohort at diagnosis

In vitro tested antibiotic	Number of isolates tested against the antibiotics (*n*)	In vitro full susceptibility (*n*, %)	In vitro moderate susceptibility (*n*, %)	In vitro resistance (*n*, %)
Beta-lactams				
Penicillin	15	13 (86.7)	2 (13.3)	0
Ampicillin	44	44 (100.0)	0	0
Amoxicillin/clavulanic acid	4	4 (100.0)	0	0
Meropenem	25	25 (100.0)	0	0
Imipenem/cilastatin	21	21 (100.0)	0	0
Fluoroquinolones				
Ciprofloxacin	14	12 (85.7)	1 (7.1)	1 (7.1)
Levofloxacin	5	5 (100.0)	0	0
Other antibiotics				
Chloramphenicol	11	11 (100.0)	0	0
Clindamycin	3	2 (66.6)	0	1 (33.3)
Erythromycin	15	15 (100.0)	0	0
Gentamicin	43	37 (86.0)	6 (14.0)	0
Rifampicin	12	12 (100.0)	0	0
Tetracycline	13	13 (100.0)	0	0
Trimethoprim/sulfamethoxazole	44	43 (97.7)	0	1 (2.3)
Vancomycin	10	10 (100.0)	0	0

### Clinical outcomes and therapeutic strategies

Clinical outcomes are detailed in Table 5. In-hospital all-cause mortality was 27.0% (17/63), and residual neurological symptoms were present in 17.5% (11/63) of cases. Admission to the ICU was necessary in 44.4% (28/63) of patients, representing a statistically significant difference between subcohorts (8/35, 22.9% vs. 20/28, 71.4%, *p* < 0.01). Brain abscess occurred in 9.5% (6/63) of cases. The median length of stay in the ward and ICU was 21.5 ± 12.0 days (18.0 ± 8.8 vs. 26.0 ± 9.0, *p* = 0.02) and 19.5 ± 12.5 days (9.0 ± 6.5 vs. 20.0 ± 8.8, *p* = 0.01), both significantly lower in the uncomplicated disease course subcohort. Only 21/46 (45./%) had long-term reliable information of sequeale available in 2023. From these patients, 13/21 (61.9%) had pesistent neurological deficits, and 7/21 (33.3%) had ongoing deterioration of health (mostly cardiovascular).

**Table 5:** Clinical outcomes of patients treated with invasive *Listeria monocytogenes* infection and characteristics of their antimicrobial and supportive therapy, by outcome subgroup

Parameter	Full cohort (*n* = 63)	Uncomplicated disease course (*n* = 35)	Complicated disease course (*n* = 28)	*p* value
Clinical outcomes (n, %)				
- In-hospital all-cause mortality (n, %) - Residual neurological symptoms (n, %) - Requirement of ICU admittance (n, %) - Occurrence of brain abscess (n, %)	17 (27.0) 11 (17.5) 28 (44.4) 6 (9.5)	0 0 8 (22.9) 0	17 (60.7) 11 (39.3) 20 (71.4) 6 (21.4)	n.a. n.a. **< 0.01** n.a.
LOS (days, median ± IQR, min-max)	21.5 ± 12.0 (7–80)	18.0 ± 8.8 (7.0–77.0)	26.0 ± 9.0 (14.0–80.0)	**0.02**
ICU LOS (days, median ± IQR, min-max)	19.5 ± 12.5 (6.0–41.0)	9.0 ± 6.5 (6.0–27.0)	20.0 ± 8.8 (6.0–41.0)	**0.01**
Temporal characteristics of antibiotic therapy (days, median ± IQR, min-max)				
- Time from admission to empirical therapy - Duration of empirical therapy - Time from admission to targeted therapy - Duration of targeted iv. therapy - Total duration of antibiotic therapy	0.0 ± 1.0 (0.0–7.0) 2.0 ± 1.0 (1.0–14.0) 3.0 ± 2.0 (0.0–19.0) 16.0 ± 10.0 (2.0–33.0) 17.0 ± 8.0 (2.0–74.0)	0.0 ± 0.0 (0.0–2.0) 3.0 ± 2.0 (1.0–14.0) 3.0 ± 2.0 (0.0–6.0) 15.0 ± 6.8 (2.0–28.0) 16.0 ± 7.0 (2.0–74.0)	0.0 ± 1.0 (0.0–7.0) 2.0 ± 1.0 (1.0–7.0) 3.0 ± 2.0 (0.0–19.0) 17.0 ± 10.0 (5.0–33.0) 20.0 ± 9.0 (6.0–35.0)	0.71 0.49 0.33 0.68 0.16
Adequacy of empirical antibiotic therapy against *Listeria* sp. (n, %)				
- Adequate - Inadequate - No data on empirical therapy	38 (60.3) 15 (23.8) 10 (15.9)	21 (60.0) 8 (22.9) 6 (5.7)	17 (60.7) 7 (25.0) 4 (14.3)	1.0 1.0 1.0
Adequate empirical antibiotic therapy (n, %)				
- Ampicillin monotherapy - Ampicillin and meropenem - Ampicillin and vancomycin - Amoxicillin monotherapy - Meropenem monotherapy - Vancomycin monotherapy	27 (42.9) 1 (1.6) 3 (4.8) 1 (1.6) 4 (6.3) 2 (3.2)	16 (45.7) 0 (0.0) 2 (5.7) 0 (0.0) 3 (8.6) 0 (0.0)	11 (39.3) 1 (3.6) 1 (3.6) 1 (3.6) 1 (3.6) 2 (7.2)	0.79 n.a. 1.0 n.a. 0.62 n.a.
Inadequate empirical antibiotic therapy (n, %)				
- Ceftriaxone monotherapy - Other antibiotic*	11 (17.5) 4 (6.3)	6 (17.1) 2 (5.7)	5 (17.9) 2 (7.1)	1.0 1.0
Targeted antibiotic therapy (n, %)				
- Ampicillin monotherapy - Ampicillin and gentamicin - Meropenem monotherapy - Ampicillin and meropenem - Meropenem and trimethoprim/sulfamethoxazole - Trimethoprim/sulfamethoxazole monotherapy - No data on targeted therapy	31 (49.2) 17 (27.0) 7 (11.1) 2 (3.2) 3 (4.8) 2 (3.2) 1 (1.6)	22 (62.9) 7 (20.0) 1 (2.9) 1 (2.9) 1 (2.9) 2 (5.7) 1 (2.9)	9 (32.1) 10 (35.7) 6 (21.4) 1 (3.6) 2 (7.1) 0 (0.0) 0 (0.0)	**0.02** 0.25 **0.03** 1.0 0.58 n.a. n.a.
Source control performed (n, %)	1 (1.6)	1 (2.9)	0 (0.0)	n.a.
Supportive therapy (n, %)				
- Vasopressor therapy - Invasive mechanical ventilation - Mannitol therapy - Red blood cell transfusion - G-CSF therapy - Systemic corticosteroid therapy	11 (17.5) 29 (46.0) 40 (63.5) 19 (30.2) 3 (4.8) 52 (82.5)	2 (5.7) 7 (20.0) 18 (51.4) 5 (14.3) 2 (5.7) 30 (85.7)	9 (32.1) 22 (78.6) 22 (78.6) 14 (50.0) 1 (3.6) 22 (78.6)	**< 0.01** **< 0.01** **0.03** **< 0.01** 0.69 0.46
Stopping corticosteroid therapy after confirmation of CNS invasive listeriosis (n, %)	11 (17.5)	7 (20.0)	4 (14.3)	0.59

CNS: central nervous system, G-CSF: granulocyte colony stimulating factor, IQR: intequartile range, ICU: intensive care unit, LOS: length of stay, n.a.: not applicable

* Cefazolin, clarithromycin, metronidazole, moxifloxacin

Characteristics of therapeutic strategies are provided in Table 5. The median duration of total antibiotic therapy was 17.0 ± 8.0 days, with a median duration of empiric therapy of 2.0 ± 1.0 days. Adequate empiric treatment against *Listeria* spp. was administered in 38/63 cases (60.3%). Inadequate empiric therapy was started at similar rates in both subcohorts (8/35, 22.9% vs. 7/28, 25.0%, *p* = 1.0). The most common adequate antibiotic choice was ampicillin monotherapy (27/63, 42.9%). Ceftriaxone monotherapy was initiated as ineffective empirical therapy in 11/63 cases (17.5%). Targeted therapy was initiated after a median of 3.0 ± 2.0 days from admission, with the median duration of intravenous therapy being 16.0 ± 10.0 days. The prevalent choices for targeted antibiotic therapy were ampicillin (31/63, 49.2%), ampicillin plus gentamicin (17/63, 27.0%), and meropenem (7/63, 11.1%). Oral switch occurred in 7.9% (5/63) of cases, with a median treatment duration of 31.5 ± 24.5 days. Survivors and those with a brain abscess received a median total duration of 28.5 ± 2.5 and 36.0 ± 6.5 days of antibiotics, respectively. Vascular reconstructive surgery for an infected femoral aneurysm was required as source control in one patient (1.6%). Systemic corticosteroid therapy was administered to 82.5% (52/63) of cases, and it was discontinued in 11/63 (17.5%) cases after invasive listeriosis was confirmed.

## Discussion

### Epidemiology of invasive listeriosis

Invasive listeriosis is a relatively rare but serious infection. In the USA, it ranks as the third most common fatal foodborne infection after *Vibrio vulnificus* and *Clostridium botulinum* [21]. Additionally, *L. monocytogenes* is the fifth most common causative agent of community-acquired acute bacterial meningitis in the USA [22, 23]. In Spain, a multicentric study of 5696 cases revealed an increasing incidence of listeriosis between 1997 and 2015, with 50% of the affected population being ≥ 65 years of age, 7% being pregnant, and 4% being newborns. Roughly half of the patients had risk factors such as malignancy (23%), diabetes mellitus (17%), and chronic liver disease (13%). The overall mortality rate was 17%, with most of the deceased being from the elderly population (68%) [24]. Emphasizing prevention and early diagnosis in populations at risk of invasive disease is of cardinal importance [4–6].

The incidence of invasive listeriosis at our center was relatively low, as we confirmed 63 cases during a 16-year period. We mostly identified cases of neurolisteriosis and isolated bacteremia, with neonatal and pregnancy-associated infections being underrepresented, possibly due to frequent referral of these patient groups to other centers. Additionally, empirical treatment without proper diagnosis might be common during pregnancy, along with an emphasis on avoiding certain foods [25].

### Clinical characteristics and diagnosis of invasive listeriosis

Results from a retrospective USA study suggest that the primary predisposing factors for neurolisteriosis are active oncohematological malignancy, post-transplantation status, and long-term corticosteroid therapy. In these patient groups, *Listeria* sp. was the most prevalent pathogen causing acute bacterial meningitis. Furthermore, chronic excessive alcohol consumption, immunosuppressive therapies, human immunodeficiency virus infection, and other comorbidities (such as diabetes mellitus, autoimmune diseases, and hereditary hemochromatosis) should also be considered, although neurolisteriosis without any known predisposing factor can occur quite frequently (36%) [14].

In our cohort, a predisposing risk factor for listeriosis could be identified in 94%, with old age, antacid therapy, chronic liver disease, diabetes mellitus, and long-term corticosteroid treatment being common. The median age in the cohort was high, and higher Charlson score was associated with complicated disease. Most clinical signs were nonspecific for the final diagnosis, but our data suggest that the absence of fever, and the appearance of focal neurological signs with symptoms of cardiorespiratory instability indicate a more severe disease. Patients with uncomplicated disease course, possibly due to lower likelihood of altered mental status, were more likely to complain of headaches and lower back pain. Cranial imaging and EEG results did not identify any localizations or morphological abnormalities highly predictive of neurolisteriosis. Overally, microbiological positivity from CSF cultures was higher than from blood cultures. While the predominance of neurolisteriosis in our cohort may explain this phenomenon, it should be emphasized that blood cultures also play a significant role in identifying bacterial invasion, as shown by previous studies [14, 26]. The use of non-culture-based microbiological tests, particularly PCR-based assays, is also likely to facilitate accurate diagnosis.

### Therapy and outcomes of invasive listeriosis

The majority of isolated *L. monocytogenes* strains were found to be highly susceptible to beta-lactams in vitro. Isolates also exhibited full in vitro susceptibility to other antibiotics, such as vancomycin, erythromycin, tetracycline, and chloramphenicol, but their clinical use should probably be avoided due to observed in vivo failures and the potential for plasmid-mediated acquired resistance [27, 28]. Among recommended antibiotics, gentamicin and trimethoprim/sulfamethoxazole showed a low prevalence for moderate susceptibility or resistance, but these drugs are primarily considered as combination or alternative agents [14]. While in vitro susceptibility of *L. monocytogenes* isolates remains high, increasing acquired resistance to several commonly used antibiotics, such as clindamycin, trimethoprim/sulfamethoxazole, and ciprofloxacin, has been observed in other studies from different geographical areas [27–29]. Knowledge of these susceptibility patterns is particularly crucial when choosing an empirical treatment regimen for immunocompromised patients. While data from our study might offer reassurance in this aspect, continued active monitoring may be necessary.

Surprisingly, 24% of all patients received empirical antibiotics that were ineffective against *Listeria* sp., although the rates were similar in the subcohorts. Literature evidence suggests that early inadequate therapy may correlate with higher mortality and a tendency for residual neurological symptoms [30]. Although meropenem exhibits excellent in vitro anti-Listeria activity, its clinical use has been associated with increased mortality in some studies [31, 32]. In our cohort, higher meropenem and lower ampicillin administration rates among patients with a complicated disease may reflect these prior findings, or the tendency of chosing broader spectrum empirical therapy for patients presenting with a more severe disease. Targeted therapy was initiated 3 days after admission on average, with the majority (76%) receiving ampicillin or an ampicillin–gentamicin combination, aligning with literature recommendations [13]. Despite a relatively extended duration of antimicrobial treatment (17 ± 8 days), only 8% of cases were switched to oral therapy. In our cohort, one patient underwent successful source control by surgery. While *Listeria* sp. associated endovascular infections are rare, they can be severe; for instance, 60% of affected patients experienced vascular rupture in a French study [33]. As expected, a significantly higher proportion of supportive therapies were needed among patients with more severe outcomes. Historically, dexamethasone therapy, often initiated in suspected acute bacterial meningitis, was believed to be terminated in cases of invasive listeriosis, as its positive effect on clinical outcomes has not been unequivocally demonstrated and, in some studies, it has been associated with increased mortality and delayed cerebral damage [10, 13, 34]. Nonetheless, a recent prospective nationwide cohort study of 162 neuroinvasive cases showed that the adjusted odds ratio of dexamethasone treatment for an unfavourable outcome was 0.4 (95%CI 0.19–0.81), providing an association with an improvement among patients with acute bacterial meningitis caused by *L. monocytogenes* [35].

### Limitations

Due to the retrospective nature of our study, recall and documentational bias is probably inevitable. Also, conducting a multicentric, prospective study would provide more accurate data collection and inclusion of a larger number of patients. We note that the universal definition of sepsis changed during 2016. Despite these limitations, we believe that our study offers a comprehensive overview of the 16-year experience with invasive listeriosis from a tertiary referral center, and could serve as a starting point for future research as the relatively rare occurrence of this infection makes it difficult to study.

## Conclusion

In our study, invasive *Listeria monocytogenes* infections were associated with high mortality and a propensity for prolonged hospitalizations and residual neurological symptoms during follow-up.

## Electronic supplementary material

Below is the link to the electronic supplementary material.

Supplementary Material 1

## Data Availability

Anonymized data of patients are available from the corresponding author on reasonable request.

## Abbreviations

CT: Computed tomography
CSF: Cerebrospinal fluid
EEG: Electroencephalography
EUCAST: European Committee on Antimicrobial Susceptibility Testing
HIV: Human immunodeficiency virus
ICU: Intensive care unit
IQR: Interquartile range
LOS: Length of stay
MRI: Magnetic resonance imaging
PCR: Polymerase chain reaction
SIRS: Systemic Inflammatory Response Syndrome
