# Supplementary material for: Clinical and microbiological characteristics and follow-up of invasive Listeria monocytogenes infection among hospitalized patients: real-world experience of 16 years from Hungary
Source: BMC Microbiol. 2024 Sep 6;24:325. doi: 10.1186/s12866-024-03478-z (PMC11378541; doi:10.1186/s12866-024-03478-z)
Supplement: Supplementary file 1 — Supplementary Material 1 [file 12866_2024_3478_MOESM1_ESM.docx]

| **Parameters** | **Reference**  **ranges** |
| --- | --- |
| Blood laboratory parameters (median±IQR, min-max)  - White blood cell count (x10^9^/l)  - Absolute neutrophil granulocyte count (x10^9^ l)  - Absolute lymphocyte count (x10^9^/l)  - Absolute monocyte count (x10^9^/l)  - Haemoglobin (g/l)  - Platelet count (x10^9^/l)  - Serum procalcitonin (ng/ml)  - Serum C-reactive protein (mg/L)  - Serum lactate dehydrogenase (IU/l)  - Serum glutamate oxaloacetate transaminase (IU/l)  - Serum glutamate pyruvate transaminase (IU/l)  - Serum gamma-glutamyl transferase (IU /l)  - Serum alkaline phosphatase (IU/l)  - Serum urea (mmol/l)  - Serum creatinine (µmol/l)  - Serum glucose (mmol/l)  - Serum albumin (g/l)  - Serum total protein (g/l)  - Serum total bilirubin (µmol/l) | 4.5‒10.0  2.5‒6.0  1.5‒3.0  0.3‒0.6  125‒165  150.0‒400.0  <0.05  <10.0  150.0‒450.0  10.0‒30.0  10.0‒40.0  10.0‒60.0  40.0‒150.0  <10.0  80.0‒120.0  3.5‒6.5  35‒55  60‒80  <17 |
| CSF laboratory parameters (median±IQR, min-max)  - CSF white blood cell count (/mm^3^)  - CSF neutrophil granulocytes (%)  - CSF lymphocytes (%)  - CSF monocytes (%)  - CSF lymphocyte/neutrophil granulocyte ratio  - CSF glucose (mmol/l)  - CSF protein (g/l) | <1  n.a.  n.a.  n.a.  n.a.  0.2‒0.6  0.6‒1.2 |

**Supplementary table 1.** Laboratory parameters assessed in the study with reference ranges at our center. n.a. = not applicable.
